# What Our Contemporaries Think of Us

**Published:** 1895-12

**Authors:** 


### What Our Contemporaries Think of Us.

*(Continued from the* Journal *for September.)*

---

[Journal of the Medical Sciences, Fort Wayne, Ind.]

We are pleased to announce that the Buffalo Medical Journal will in a few weeks have reached fifty years, the *golden age.* We congratulate the editor and publishers on the success of this journal. Few, very few, indeed, of the medical journals of this country have reached the age of fifty years and it gives us great pleasure in calling the attention of the profession to the fact that the Buffalo Medical Journal has attained the *golden age.* We extend to the editor our hearty congratulations and trust the Journal may prosper in the future as it has in the past. It is one among our most valued exchanges and we always welcome it to our table.

---

[Atlanta Medical and Surgical Journal.]

The August issue of the Buffalo Medical Journal is the jubilee number, beginning the second half-century of its existence. Besides some excellent medical and surgical articles, it contains a full history of the Medical Journal, from its organisation in 1845 by the elder Austin Flint through all its administrations down to the present time. The same article, which is by the present able editor, Dr. William Warren Potter, deals with other medical matters in the City of Buffalo for the same period. We congratulate our esteemed exchange upon its past history and wish it a long and prosperous future.

---

[St. Louis Medical and Surgical Journal.]

The Buffalo Medical Journal celebrated its golden jubilee by issuing a large and handsome number for its August issue. Established in 1845, by Austin Flint, it has continued to prosper, and bids fair to excel its previous record under the fostering care of its present editors. Our younger cotemporary, for it is two years our junior, shows no sign of decrepitude, despite the fifty years which have accumulated on its shoulders, but it is rather a bright example of youth renewed and vigor undiminished.

---

[Memphis Medical Monthly ]

The BUFFALO MEDICAL JOURNAL, in celebrating by gala number its fiftieth birthday, has proven, by its survival and constant growth during this period, that it is one of the fittest. May it continue, as now, in the front rank of medical journalism and live to celebrate many more anniversaries.

---

[Richmond Journal of Practice.]

The BUFFALO MEDICAL JOURNAL for August, 1895, comes to us in a handsome new dress and greatly enlarged. In addition to more than its usual amount of scientific original papers, medical items and editorials, there is a special article of forty-eight pages from the pen of one of its present talented editors, Dr. William Warren Potter, giving in his own peculiar, graphic style a historical reminiscence of medical journalism in Buffalo during the last fifty years, together with its medical colleges, hospitals and medical societies. A view of Buffalo in 1845, with its then 30,000 population, contrasted with the present beautiful composite picture in 1895 and with its 360,000 inhabitants, gives some idea of the material advances of this lovely city. Dr. Potter claims to give only a résumé of the salient events of the past fifty years of its medical history. In doing this he gives the names of those physicians connected with the formation and progress of its colleges, hospitals and local societies down to the present time Many finely executed engravings of hospitals and other medical buildings serve to connect the past with the present as well as to forecast the future of this progressive medical center.

The founder, editor and owner of this sterling medical journal was the great medical teacher and author, Dr. Austin Flint. The present editors are Drs. Thomas Lothrop and William Warren Potter, who are ably assisted by an associate staff, consisting of Drs. William C. Krauss, John A. Miller, Ernest Wende, James W. Putnam, John Parmenter and Alvin A. Hubbell. The JOURNAL has witnessed nearly all the improvements in medicine and surgery that are valuable today, and it has reason to triumph over its long life, so full of progress and scientific research. With the August number the present able management have signalled its appreciation of the support from all who have had a share in its life by "donning new garments, manifesting new energy, increasing the number of its pages," and in a number of other ways adding new features which cannot fail to continue it in the foremost ranks of the medical periodicals of this or any other country. May the BUFFALO MEDICAL JOURNAL live to celebrate its centennial and share the honors of medical progress in the fifty years to come. To its present corps, the *Richmond Journal of Practice* extends its heartiest congratulations.

---

[Columbus Medical Journal.]

The jubilee number of the BUFFALO MEDICAL JOURNAL is a sockdolager. Our friend, Dr. Potter, spread himself and astonished his most sanguine friends, and came down the home stretch flying, distancing many of his competitors in the first heat. The JOURNAL, as usual, was especially good, in addition to the interesting history embracing a period of fifty years, between the inception of the JOURNAL by the distinguished Austin Flint and the present. We wish the JOURNAL and its enterprising editors another half century's success that will not only equal, but excel the one just past.

---

[Occidental Medical Times.]

The August issue of the BUFFALO MEDICAL JOURNAL completes the fiftieth year of its publication, and the JOURNAL'S semi-centennial has been celebrated by the issue of a jubilee number, and the permanent increase of its reading-matter from sixty-four to eighty pages. The special issue is handsomely printed and profusely illustrated. Naturally, the most interesting feature of it is the retrospect of Buffalo's medical history during the past fifty years. Herein is graphically detailed the progress of the city and its medical institutions during the years in which Buffalo, from a city of 30,000 inhabitants has steadily increased to one of 300,000. The JOURNAL was founded in 1845 by Austin Flint, and has since numbered amongst its editors many who have made their mark in the medical history of the great State of New York.

---

[Canada Medical Record, Montreal.]

We cannot lay down the August number of the BUFFALO MEDICAL JOURNAL, which is its jubilee number, and which we have just perused, without expressing our admiration for the enterprise of its editors, Drs. Thomas Lothrop and William Warren Potter. It was founded in 1845 by Dr. Austin Flint, and has consequently completed its fiftieth year. The jubilee number, besides containing many very able scientific articles, is profusely illustrated with engravings of the Buffalo hospitals. We wish our bright and newsy contemporary as much success in the future as it has attained in the past. It has always been among the most welcome of our exchanges.

[Medical Review, St. Louis.]

The jubilee number of the BUFFALO MEDICAL JOURNAL is before us; with it this journal enters upon its fifty-first year of existence. The quality of the JOURNAL is too well known to need pointing out. Besides several valuable articles the jubilee number contains the history of the JOURNAL, which is, at the same time, the history of the medical profession of Buffalo. Some of the most eminent and best known men in the American medical profession have been the editors of the JOURNAL, of which we may mention Dr. Austin Flint, Dr. Sanford B. Hunt, Dr. Austin Flint, Jr., Dr. Julius F. Miner, etc. The BUFFALO MEDICAL JOURNAL may be proud of its history and the men that have been and are connected with its management. We hope that it will continue to prosper for the benefit of the medical profession and to the honor and pride of the City of Buffalo.

---

[Journal of Practical Medicine, New York.]

The semi-centennial number of the BUFFALO MEDICAL JOURNAL, August, is at hand. It is a handsome number of 130 pages, and is made especially interesting by a large number of illustrations. The most entertaining article in the number is contributed by the present editor, Dr. William Warren Potter, and occupies nearly fifty pages. Dr. Potter has heretofore received our congratulations, and we extend them again, for it is an honor, which will come to but few of us, to celebrate the fiftieth anniversary of the journal we edit.

---

[Dental Practitioner and Advertiser, Buffalo, N. Y.]

That excellent and sterling monthly, the BUFFALO MEDICAL JOURNAL, recently celebrated its semi-centennial. It was established by Dr. Austin Flint in 1845, and it has occupied a front rank in professional literature from that day to this, but never was it more truly representative of the profession, or more prosperous than today, under the editorship of Dr. Thomas Lothrop and Dr. William Warren Potter. Its semi-centennial number was worthy its reputation, and whether in the value of its contents or the beauty of its typography and illustrations, it was a memorable issue. May it continue to flourish until it shall reach its centennial, and then but enter upon a new era of prosperity.

---

[Quarterly Medical Journal, Sheffield, Eng.]

The BUFFALO MEDICAL JOURNAL has completed its jubilee. It was started in 1845, when Buffalo contained only 45,000 inhabitants; now that city numbers a population of upwards of 360,000. Austin Flint was editor, founder and also proprietor of this sturdy representative of what may be called provincial medical journalism in the United States. On the editorial roll is found also the name of Austin Flint, Jr. In the early days of the JOURNAL Dr. Frank Hastings Hamilton recorded in its pages his surgical clinics and fracture tables, from which sprang the material upon which he founded his authoritative and classical work on fractures and dislocations. The jubilee number (August, 1895,) is before us, and besides many very excellent articles on a variety of subjects, it contains much readable and interesting information as to the men who made the journal what it is.

